# Cell-type-specific nicotinic input disinhibits mouse barrel cortex during active sensing

**DOI:** 10.1016/j.neuron.2020.12.018

**Published:** 2021-03-03

**Authors:** Célia Gasselin, Benoît Hohl, Arthur Vernet, Sylvain Crochet, Carl C.H. Petersen

**Affiliations:** 1 Laboratory of Sensory Processing, Brain Mind Institute, Faculty of Life Sciences, Ecole Polytechnique Fédérale de Lausanne (EPFL), Lausanne, Switzerland

**Keywords:** acetylcholine, neocortex, GABAergic inhibition, disinhibition, whisker sensorimotor processing, two-photon microscopy, whole-cell recording, membrane potential, optogenetics, nicotinic receptors

## Abstract

Fast synaptic transmission relies upon the activation of ionotropic receptors by neurotransmitter release to evoke postsynaptic potentials. Glutamate and GABA play dominant roles in driving highly dynamic activity in synaptically connected neuronal circuits, but ionotropic receptors for other neurotransmitters are also expressed in the neocortex, including nicotinic receptors, which are non-selective cation channels gated by acetylcholine. To study the function of non-glutamatergic excitation in neocortex, we used two-photon microscopy to target whole-cell membrane potential recordings to different types of genetically defined neurons in layer 2/3 of primary somatosensory barrel cortex in awake head-restrained mice combined with pharmacological and optogenetic manipulations. Here, we report a prominent nicotinic input, which selectively depolarizes a subtype of GABAergic neuron expressing vasoactive intestinal peptide leading to disinhibition during active sensorimotor processing. Nicotinic disinhibition of somatosensory cortex during active sensing might contribute importantly to integration of top-down and motor-related signals necessary for tactile perception and learning.

## Highlights

• Acetylcholine is released in the mouse barrel cortex during active whisker sensing
• Acetylcholine depolarizes inhibitory cells expressing vasoactive intestinal peptide
• Excitation of vasoactive intestinal peptide-expressing neurons causes disinhibition
• Cholinergic-driven disinhibition could gate sensorimotor integration and plasticity

## Teaser

Gasselin et al. report that acetylcholine released in the mouse barrel cortex during active whisker sensing selectively excites a class of inhibitory neurons characterized by expression of vasoactive intestinal peptide through nicotinic receptors causing disinhibition of sensory processing in nearby excitatory neurons.

## Introduction

Neocortical microcircuits are composed of synaptically connected local networks of neurons, which send and receive various long-range inputs. The vast majority of synapses in the neocortex use either glutamate or GABA as a neurotransmitter packaged into synaptic vesicles, which are released at presynaptic specializations in response to action potential firing. Ionotropic receptors respond rapidly to released glutamate and GABA evoking fast postsynaptic potentials. However, ionotropic serotonin and acetylcholine receptors are also expressed in various types of cells in the neocortex, and an important challenge is to unravel their functional roles in cortical computations.

*In vitro* in brain slices, activation of ionotropic nicotinic acetylcholine receptors in response to puff application of exogenous acetylcholine or optogenetic stimulation of cholinergic axons has been found to robustly depolarize a class of neocortical non-fast-spiking GABAergic neurons expressing vasoactive intestinal peptide (VIP) (Arroyo et al., 2012; Férézou et al., 2002; Gulledge et al., 2007; Lee et al., 2010; Porter et al., 1999). Furthermore, measured *in vivo* through calcium imaging, pharmacological blockade of nicotinic receptors in mouse visual cortex reduced running-related excitation of VIP neurons (Fu et al., 2014). VIP neurons have been proposed to play a predominantly disinhibitory role in neocortical circuit function *in vivo* (Karnani et al., 2016; Lee et al., 2013; Pi et al., 2013). VIP neurons prominently inhibit somatostatin-expressing (SST) neurons and, to a somewhat lesser extent, parvalbumin-expressing (PV) neurons (Karnani et al., 2016; Lee et al., 2013; Pfeffer et al., 2013; Pi et al., 2013). SST and PV neurons, in turn, are, respectively, thought to inhibit primarily the distal and proximal aspects of the somatodendritic axes of excitatory pyramidal neurons (DeFelipe et al., 2013; Kepecs and Fishell, 2014; Tremblay et al., 2016). The inhibition of SST and PV neurons by VIP neurons is therefore likely to disinhibit nearby excitatory pyramidal neurons. Disinhibition may play important roles in cortical function including gating of sensorimotor transformation (Sachidhanandam et al., 2016), integration of sensory and motor signals (Gentet et al., 2012; Lee et al., 2013), and enhancing synaptic plasticity (Fu et al., 2015; Letzkus et al., 2011; Williams and Holtmaat, 2019).

In this study, we investigated functional roles of VIP neurons in the primary whisker somatosensory barrel cortex (wS1), a cortical region important for processing whisker-related sensorimotor information in mice (Bosman et al., 2011; Diamond et al., 2008; Feldmeyer et al., 2013; Petersen, 2019). Mice actively scan their immediate environment by moving their facial whiskers back and forth at high frequency (∼10 Hz). During such active sensing mice can palpate objects to examine their location, shape, and texture. In order for the mouse to generate accurate tactile percepts, whisker sensory input needs to be integrated with dynamic motor signals. Interestingly, active whisker sensing (whisking) is accompanied by a prominent change in brain state and sensory processing compared to adjacent epochs of quiet wakefulness (Crochet and Petersen, 2006; Ferezou et al., 2007; Hentschke et al., 2006; Poulet and Petersen, 2008). Presumably, the neuronal circuits of wS1 are moved into a state favorable for analyzing actively acquired sensory information during whisking. Previous studies have found that VIP neurons in wS1 are excited during whisking (Lee et al., 2013; Yu et al., 2019) and acetylcholine increases in wS1 during whisking (Eggermann et al., 2014). Cholinergic activation of nicotinic receptors on VIP neurons, in addition to input from motor cortex (Lee et al., 2013), could thus contribute to their excitation during whisking, which in turn might promote disinhibition of wS1 during active sensorimotor processing. Here, we investigate this hypothesis through pharmacological and optogenetic manipulations combined with whole-cell membrane potential recordings targeted through two-photon microscopy to five specific cell types in layer 2/3 of wS1 in awake head-restrained mice during quantified whisker-related behavior (Margrie et al., 2003; Petersen, 2017).

## Results

### Nicotinic input selectively depolarizes VIP neurons during whisking

**Figure 1:**
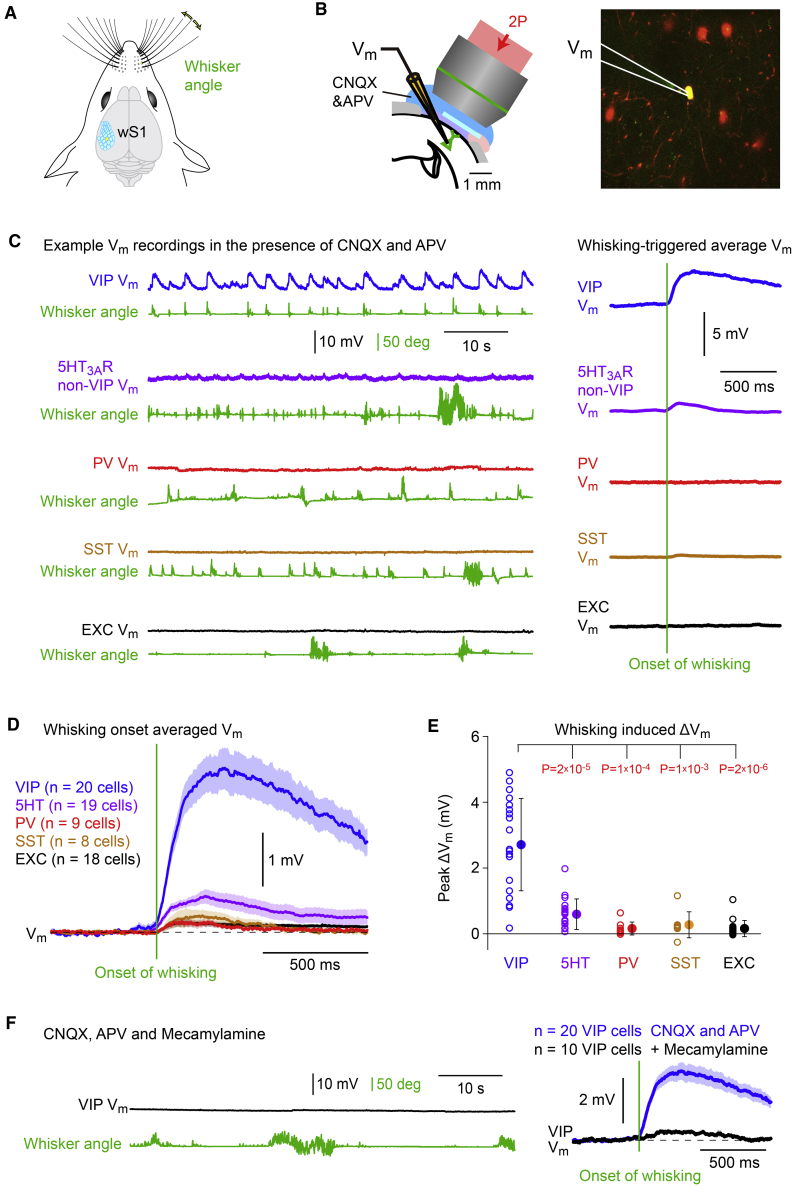
Vasoactive intestinal peptide-expressing neurons in wS1 are selectively depolarized during whisking by a non-glutamatergic input (A) The primary somatosensory barrel cortex (wS1) processes sensory information related to whisker tactile sensory perception with a well-defined somatotopic map. (B) In this study, we made whole-cell membrane potential (V_m_) recordings from fluorescently labeled genetically defined neuronal cell classes in wS1 targeted through two-photon imaging. In many experiments, we pharmacologically blocked ionotropic glutamate receptors by the presence of CNQX and APV in both the bath solution and the agarose gel stabilizing the cortex. (C) Example V_m_ recordings in the presence of CNQX and APV from different genetically defined classes of layer 2/3 wS1 cortical neurons during quantification of whisker angle (green trace). Most neurons show little fluctuation in V_m_, but the example VIP neuron appears to depolarize during whisking, and by averaging V_m_ aligned to the initiation of each whisking bout a clear depolarization is observed in this example VIP cell, but not in the other example cells from different cell classes (right). (D) Computed across the population of neurons recorded in the presence of CNQX and APV in layer 2/3 of wS1, VIP neurons depolarized prominently upon the initiation of whisking, whereas there was little impact upon the V_m_ of 5HT3AR-expressing-non-VIP neurons (5HT), parvalbumin-expressing neurons (PV), somatostatin-expressing neurons (SST), and unlabeled excitatory neurons (EXC). Grand-average V_m_ across cells (thick line) plotted together with SEM shading. (E) Each open circle quantifies the peak change in V_m_ in the presence of CNQX and APV upon initiation of whisking for each recorded neuron, color-coded according to cell type. The filled circles and error bars indicate mean ± SD. Wilcoxon rank-sum test with Bonferroni correction: VIP versus 5HT, p = 2 × 10^−5^; VIP versus PV, p = 1 × 10^−4^; VIP versus SST, p = 1 × 10^−3^; VIP versus EXC, p = 2 × 10^−6^. (F) An example V_m_ recording from a VIP neuron in the presence of CNQX, APV, and mecamylamine to block AMPA, NMDA, and nicotinic acetylcholine receptors (left). In this example recording, the V_m_ shows only small fluctuations, even during intense whisking (left). Averaged across all initiations of whisking for each neuron, and then further averaged across all recorded neurons, there was little modulation of V_m_ at whisking onset when both ionotropic glutamate receptors and nicotinic receptors were pharmacologically blocked (right). The thick black trace shows the grand average V_m_ across n = 10 VIP cells in the presence of CNQX, APV, and mecamylamine, and the shading shows SEM. The thick blue trace shows the grand average V_m_ across n = 20 VIP cells in the presence of CNQX and APV (identical to that shown in D), and the shading shows SEM. See also Figure S1.

We targeted whole-cell recordings to the C2 whisker representation in wS1 of awake head-restrained mice, and quantified C2 whisker angle through high-speed videography (Figure 1A). Specific genetically defined classes of neurons expressing tdTomato (Madisen et al., 2010; Taniguchi et al., 2011) or GFP (Gong et al., 2003; Lee et al., 2010) were visualized through *in vivo* two-photon microscopy allowing membrane potential (V_m_) recordings from sparsely labeled neuronal populations (Figure 1B). In order to reveal any neocortical activity that does not depend upon the main excitatory neurotransmitter glutamate, we locally blocked ionotropic glutamate receptors by application of CNQX and APV to block AMPA and NMDA receptors, respectively. The CNQX and APV was included in the Ringer’s solution and within the agarose overlying the craniotomy, likely completely blocking all cortical ionotropic glutamate receptors within ∼1 mm of the craniotomy. Under these conditions, V_m_ in most types of neurons was relatively stable with only relatively small dynamic fluctuations (Figure 1C). VIP neurons in contrast typically displayed obvious membrane potential depolarizations, which appeared to relate to bouts of active whisking (Figure 1C). Aligned to the onset of whisking, VIP neurons depolarized by 2.7 ± 1.4 mV (n = 20 cells), 5HT3A-non-VIP neurons depolarized by 0.6 ± 0.5 mV (n = 19 cells), PV neurons depolarized by 0.2 ± 0.2 mV (n = 9 cells), SST neurons depolarized by 0.3 ± 0.4 mV (n = 8 cells), and unlabeled excitatory neurons depolarized by 0.2 ± 0.2 mV (n = 18 cells) (Figures 1D and 1E). The whisking-related depolarization of VIP neurons during glutamate receptor blockade was significantly larger than for any of the other layer 2/3 cell classes studied here (Kruskal-Wallis test, p = 1 × 10^−9^; VIP versus other cell types, Wilcoxon rank-sum test with Bonferroni correction, p < 1 × 10^−3^) (Figure 1E). This depolarization of VIP neurons during whisking was almost entirely blocked by application of the nicotinic receptor antagonist mecamylamine (whisking onset depolarization: 0.3 ± 0.2 mV, n = 10 VIP cells; CNQX and APV versus CNQX, APV and mecamylamine, Wilcoxon rank-sum test, p = 5 × 10^−5^) (Figure 1F). Stronger whisking was accompanied by larger depolarization (Figures S1A and S1B). In agreement with a previous study (Eggermann et al., 2014), we found that GCaMP6s expressed in cholinergic neurons of the nucleus basalis increased fluorescence during whisking (Figure S1C). Among the various types of neurons in layer 2/3 of wS1, VIP neurons therefore appear to be the main target of nicotinic input during active whisker sensing.

### Whisker deflection can depolarize VIP neurons without glutamatergic synaptic input

**Figure 2:**
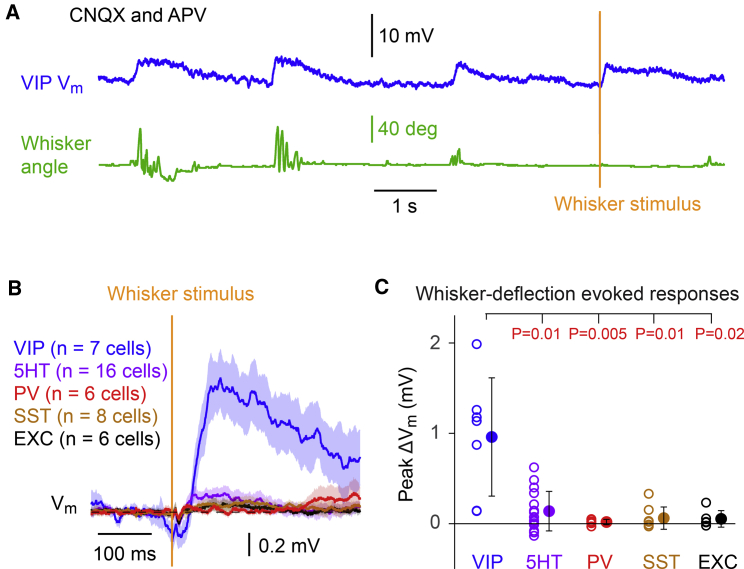
Whisker stimulation specifically depolarizes layer 2/3 VIP neurons in the presence of glutamate receptor blockers (A) An example V_m_ recording of a VIP neuron in the presence of CNQX and APV to block glutamatergic synaptic excitation during an epoch including bouts of self-initiated whisking and the delivery of a whisker stimulus without actively induced whisker movements. (B) Measured in the presence of CNQX and APV, VIP neurons depolarized in response to whisker deflection, whereas there was no obvious sensory-evoked response in 5HT3AR-expressing-non-VIP neurons (5HT), parvalbumin-expressing neurons (PV), somatostatin-expressing neurons (SST), or unlabeled excitatory neurons (EXC). Grand-average V_m_ across cells (thick line) plotted together with SEM shading. (C) The peak depolarization of VIP neurons in response to whisker deflection was significantly larger than for other layer 2/3 cell classes in the presence of CNQX and APV. Each open circle represents the data from a single cell, and filled circles with error bars indicate mean ± SD. Wilcoxon rank-sum test with Bonferroni correction: VIP versus 5HT, p = 0.01; VIP versus PV, p = 0.005; VIP versus SST, p = 0.01; VIP versus EXC, p = 0.02.

Having found that neocortical VIP neurons can depolarize during whisking through nicotinic receptor activation, we next wondered whether whisker stimulation might be able to evoke sensory responses in layer 2/3 VIP neurons of wS1 even in the absence of glutamatergic synaptic transmission (Figure 2). We attached a small metal particle to the C2 whisker and delivered 1-ms magnetic pulses to briefly deflect it. Whisker movements were monitored through high-speed filming, as before, and we specifically analyzed whisker stimuli that were not accompanied by active whisking (Figure 2A). During blockade of glutamate receptors by CNQX and APV, whisker stimulation evoked a small depolarizing response in VIP neurons with a latency of 53 ± 11 ms (n = 5 cells). We recorded across the various cell types in layer 2/3 of wS1 during ionotropic glutamate receptor blockade finding that VIP neurons depolarized most strongly in response to whisker deflection (Figures 2B and 2C). VIP neurons were depolarized by 1.0 ± 0.7 mV (n = 7 cells), 5HT3A-non-VIP neurons were depolarized by 0.1 ± 0.2 mV (n = 16 cells), PV neurons were depolarized by 0.0 ± 0.0 mV (n = 6 cells), SST neurons were depolarized by 0.1 ± 0.1 mV (n = 8 cells), and unlabeled excitatory neurons were depolarized by 0.0 ± 0.1 mV (n = 6 cells) (Figure 2C). The sensory-evoked response during glutamate receptor blockade was significantly larger in VIP neurons compared to the other layer 2/3 cell classes studied here (Kruskal-Wallis test, p = 6 × 10^−3^; VIP versus other cell types, Wilcoxon rank-sum test with Bonferroni correction, p < 0.02) (Figure 2C).

### Optogenetic cholinergic stimulation selectively depolarizes VIP neurons

**Figure 3:**
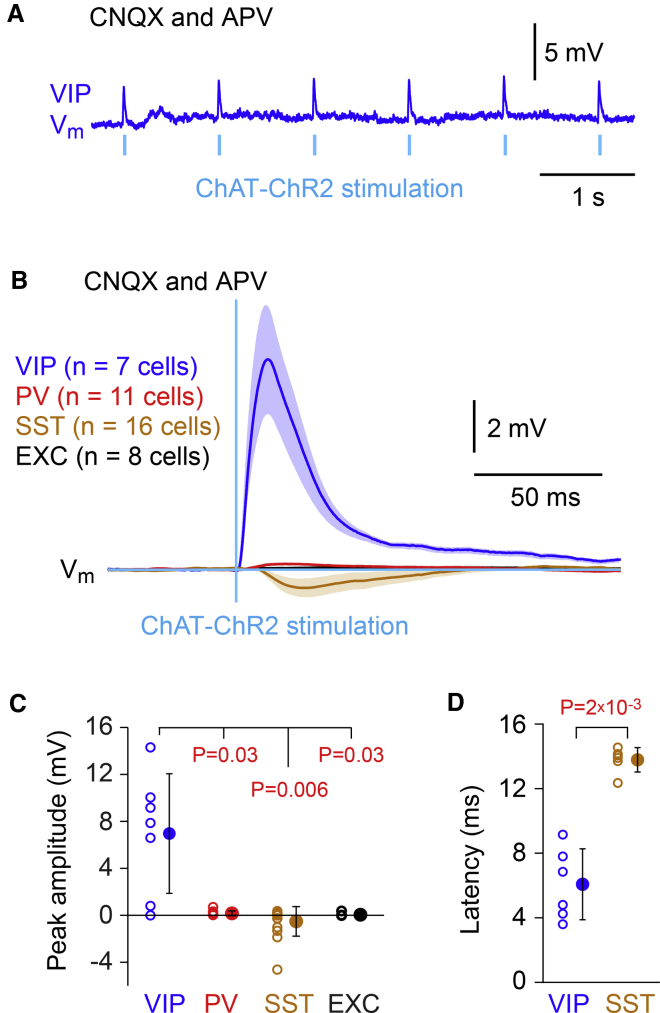
Optogenetic cholinergic stimulation specifically depolarizes VIP neurons in layer 2/3 of wS1 (A) An example V_m_ recording from a VIP neuron during optogenetic cholinergic stimulation in the presence of CNQX and APV to block glutamatergic excitation. (B) Grand average cell-type-specific responses to optogenetic cholinergic stimulation, including VIP, PV, SST, and EXC neurons. VIP neurons depolarized strongly and rapidly in response to a brief cholinergic optogenetic stimulation. PV and excitatory neurons were largely unaffected by cholinergic stimulation, but SST neurons on average appeared to hyperpolarize after a delay. Grand-average V_m_ across cells (thick line) plotted together with SEM shading. (C) VIP neurons depolarized significantly more strongly in response to optogenetic cholinergic stimulation than other cell types in wS1. Each open circle represents the data from a single cell, and filled circles with error bars indicate mean ± SD. Wilcoxon rank-sum test with Bonferroni correction: VIP versus PV, p = 0.03; VIP versus SST, p = 0.006; VIP versus EXC, p = 0.03. (D) The hyperpolarizing inhibition observed in some (6 out of 16) SST neurons of wS1 in response to optogenetic cholinergic stimulation was significantly delayed compared to the depolarization in VIP neurons (VIP versus SST, Wilcoxon rank-sum test, p = 0.002). Each open circle represents the data from a single cell, and filled circles with error bars indicate mean ± SD.

To further test the hypothesis that VIP neurons selectively receive nicotinic inputs, we delivered brief blue light flashes to wS1 of transgenic ChAT-ChR2 mice to optogenetically evoke release of acetylcholine (Zhao et al., 2011). These mice were additionally crossed with the mice expressing tdTomato in specific classes of GABAergic neurons, allowing targeted whole-cell recordings, as before. In the presence of CNQX and APV to block ionotropic glutamate receptors, a rapid depolarization was prominent in VIP neurons (Figure 3A). In contrast PV and excitatory layer 2/3 neurons showed little or no response to the optogenetic stimulation, but there appeared to be a small delayed hyperpolarization in some SST neurons (Figure 3B). The peak V_m_ response amplitude evoked by the optogenetic stimulus across cell types was: VIP, 7.0 ± 5.0 mV (n = 7 cells); PV, 0.2 ± 0.2 mV (n = 11 cells); SST, −0.5 ± 1.0 mV (n = 16 cells), and EXC, 0.0 ± 0.1 mV (n = 8 cells) (Figure 3C). The peak V_m_ response was significantly larger for VIP neurons compared to the other cell types (Kruskal-Wallis test, p = 7 × 10^−4^; VIP versus other cell types, Wilcoxon rank-sum test with Bonferroni correction, p < 0.03) (Figure 3C). As a population, the SST neurons did not significantly hyperpolarize (Wilcoxon signed-rank test, p = 0.4), but six out of 16 recorded SST neurons hyperpolarized significantly compared to shuffled data. The latency of the hyperpolarizing response in the significantly modulated SST neurons (n = 6 cells) was found to be significantly longer than the depolarizing response in the significantly modulated VIP neurons (n = 6 cells) (Wilcoxon rank-sum test, p = 0.002) (Figure 3D). Previous work has shown that VIP neurons prominently inhibit SST neurons (Lee et al., 2013; Pfeffer et al., 2013; Pi et al., 2013), and the delayed hyperpolarization observed here could thus result from cholinergic excitation of VIP neurons inhibiting nearby SST neurons.

### Disinhibition of excitatory neurons in wS1 by VIP neurons during active whisker sensation

**Figure 4:**
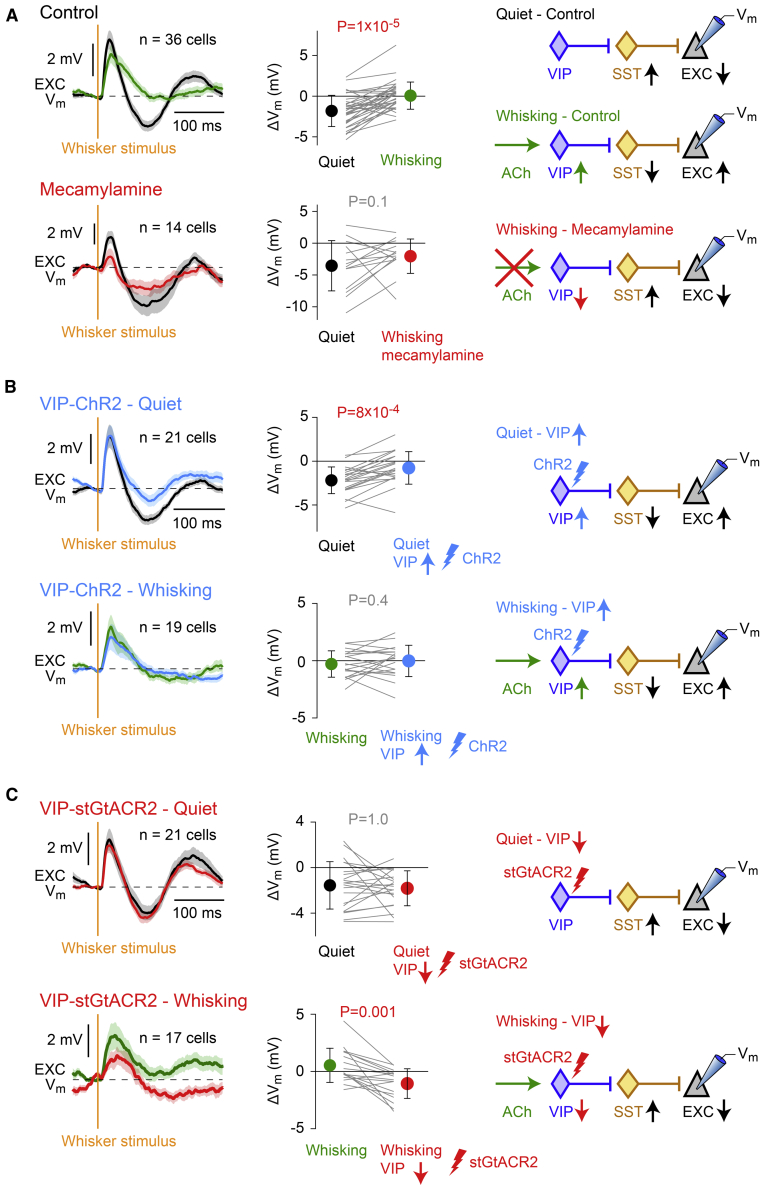
VIP neurons likely contribute to nicotinic disinhibition of wS1 during whisking (A) Whisker deflection evoked a dynamic V_m_ response in wS1 excitatory neurons including an early excitation and a later secondary inhibition. Note that CNQX and APV were not applied. When the whisker was deflected during a period of whisking, the response was smaller in peak amplitude but also longer lasting with less hyperpolarization (control: quiet versus whisking, Wilcoxon signed-rank test, p = 1 × 10^−5^). Whisking is thus associated with a late disinhibition of wS1 in response to whisker deflection. However, application of mecamylamine to block nicotinic acetylcholine receptors prevented this whisking induced disinhibition (mecamylamine: quiet versus whisking, Wilcoxon signed-rank test, p = 0.1). Nicotinic excitation of VIP neurons might thus drive disinhibition of wS1 during whisking. (B) Optogenetic activation of VIP neurons expressing ChR2 promotes disinhibition in wS1 excitatory neurons in quiet trials but has little effect upon whisking trials (quiet trials: control versus VIP-ChR2 stimulation, Wilcoxon signed-rank test, p = 8 × 10^−4^; whisking trials: control versus VIP-ChR2 stimulation, Wilcoxon signed-rank test, p = 0.4). Note that CNQX and APV were not applied. (C) Optogenetic inhibition of VIP neurons expressing the light-activated chloride channel stGtACR2 impacted whisker-deflection evoked responses recorded in wS1 excitatory neurons during whisking epochs but not during quiet periods (quiet trials: control versus VIP-stGtACR2 inhibition, Wilcoxon signed-rank test, p = 1.0; whisking trials: control versus VIP-stGtACR2 inhibition, Wilcoxon signed-rank test, p = 0.001). Note that CNQX and APV were not applied. The traces on the left in (A), (B), and (C) show grand-average V_m_ across cells (thick line) plotted together with SEM shading. The graphs in the middle column in (A), (B), and (C) show the results of individual cells (grey lines). The filled circles with error bars indicate mean ± SD. See also Figure S2.

The prominent nicotinic input during active whisking that selectively depolarizes VIP neurons in layer 2/3 (Figure 1) could mediate disinhibition of excitatory pyramidal neurons in wS1 through suppressing activity in SST and PV neurons (Gentet et al., 2012; Lee et al., 2013; Pfeffer et al., 2013; Pi et al., 2013). Here, we therefore made whole-cell recordings from layer 2/3 excitatory neurons examining V_m_ responses to magnetic whisker deflections during quiet and whisking periods, while applying mecamylamine to inhibit nicotinic receptors (Figure 4A), optogenetically stimulating VIP neurons (Figure 4B) or optogenetically inhibiting VIP neurons (Figure 4C). In these experiments, we did not apply CNQX and APV, thus leaving excitatory synaptic transmission intact.

We first measured the whisker-deflection evoked response under control conditions separating between trials in which the mouse was not moving its whisker (quiet trials) and trials in which the mouse was actively whisking (whisking trials) (Figure 4A). The early depolarizing sensory-evoked response was reduced in amplitude during active whisking (Figure 4A), consistent with previous work (Crochet and Petersen, 2006; Ferezou et al., 2007; Hentschke et al., 2006; Yamashita et al., 2013). The reduced amplitude of the early evoked sensory response during whisking is thought to result from short-term synaptic depression of thalamocortical input to wS1 (Castro-Alamancos and Oldford, 2002; Gil et al., 1997), induced by the high firing rates of thalamic neurons during whisking (Moore et al., 2015; Poulet et al., 2012; Urbain et al., 2015). Interestingly, in addition, we found that a late secondary sensory-evoked hyperpolarization, which was evident during quiet trials, was reduced during whisking trials (quiet versus whisking trials, n = 36 neurons, Wilcoxon signed-rank test, p = 1 × 10^−5^) (Figure 4A). The late inhibition could be caused by late/prolonged firing of SST neurons in response to whisker deflection as recently reported (Yu et al., 2019). Prolonged firing of PV neurons in response to whisker deflection (Gentet et al., 2012; Sachidhanandam et al., 2016) could also contribute to the late inhibition of the nearby excitatory neurons. Our results are consistent with the hypothesis that acetylcholine released during whisking excites VIP neurons, which in turn inhibits SST neurons (and perhaps also PV neurons), thus disinhibiting excitatory neurons, removing the late inhibitory phase of the sensory-evoked response. When mecamylamine was applied to the cortex, the late inhibition was present in both quiet and whisking trials (quiet versus whisking trials, n = 14 neurons, Wilcoxon signed-rank test, p = 0.1) (Figure 4A). Blocking nicotinic receptors reduces whisking-related depolarization of VIP neurons (Figure 1F), and thus SST (and perhaps PV) neurons might be less inhibited, allowing them to evoke the late inhibition in excitatory neurons even during whisking trials, as observed (Figure 4A).

We next carried out optogenetic manipulations to test more specifically for the involvement of VIP neurons in controlling the late inhibitory phase of sensory processing. VIP neurons expressing ChR2 (Boyden et al., 2005; Nagel et al., 2003) were optogenetically stimulated through application of continuous blue light starting 200 ms before whisker deflection and continuing for 300 ms after the whisker stimulus (Figure S2A). The optogenetic excitation of VIP neurons reduced the late inhibition in quiet trials (light versus no light quiet trials, n = 21 neurons, Wilcoxon signed-rank test, p = 8 × 10^−4^) without affecting whisking trials (light versus no light whisking trials, n = 19 neurons, p = 0.4) (Figure 4B). During whisking trials, the VIP neurons are already excited, and thus additional optogenetic stimulation may not have an important impact. During quiet trials, VIP firing is low (Lee et al., 2013), and the optogenetic excitation of VIP neurons may have an important impact suppressing SST neurons (and perhaps PV neurons) and thus removing the late inhibitory phase of the sensory-evoked response (Figure 4B). On the other hand, optogenetic inhibition of VIP neurons expressing stGtACR2 (Mahn et al., 2018) had the converse effect (Figure 4C). Inhibiting VIP neurons (Figure S2B) had little impact upon sensory-evoked responses in quiet trials (light versus no light quiet trials, n = 21 neurons, Wilcoxon signed-rank test, p = 0.9), consistent with an already low excitation of VIP neurons in this behavioral state, giving rise to only a small effect of inhibiting these neurons. Optogenetically inhibiting VIP neurons during whisking trials caused the emergence of a late inhibitory response to whisker stimulation (light versus no light quiet trials, n = 17 neurons, Wilcoxon signed-rank test, p = 0.001) (Figure 4C). This would be consistent with an optogenetic suppression of the whisking-related excitation of VIP neurons, allowing SST neurons (and perhaps PV neurons) to drive the late inhibitory response.

## Discussion

Our V_m_ data in layer 2/3 of wS1 suggest that a prominent nicotinic input selectively depolarizes VIP neurons during whisking and promotes disinhibition of excitatory pyramidal neurons during active sensorimotor processing. Such disinhibition might be important in wS1 for integration of motor-related signals during whisking (Gentet et al., 2012; Lee et al., 2013) and is also likely to play prominent roles in controlling cortical synaptic plasticity (Fu et al., 2015; Williams and Holtmaat, 2019).

Acetylcholine is likely to play diverse roles in controlling cortical function, many of which have previously been suggested to be mediated by slower neuromodulatory actions of acetylcholine acting via G-protein-coupled receptors. Indeed, previous work in wS1 found that cholinergic signaling via metabotropic receptors during active whisking contributes to regulating cortical state by suppressing the slow spontaneous V_m_ fluctuations found during quiet wakefulness (Eggermann et al., 2014; Meir et al., 2018). This could, at least in part, be mediated by a whisking-related activation of muscarinic receptors acting to excite deep layer SST neurons (Muñoz et al., 2017), with likely further contributions mediated by presynaptic inhibition of neurotransmitter release (Eggermann and Feldmeyer, 2009; Kruglikov and Rudy, 2008), as well as a muscarinic hyperpolarization of some excitatory neurons (Eggermann and Feldmeyer, 2009; Gulledge and Stuart, 2005). More generally, metabotropic actions of acetylcholine in cortex have been suggested to contribute to controlling brain states (Buzsaki et al., 1988; Lee and Dan, 2012; Metherate et al., 1992), enhancing attention (Harris and Thiele, 2011; Herrero et al., 2008), reward signaling (Chubykin et al., 2013), learning (Bakin and Weinberger, 1996), and cortical plasticity (Kilgard and Merzenich, 1998). Here, we characterized a prominent ionotropic effect of acetylcholine acting on nicotinic receptors to selectively depolarize a specific class of GABAergic neurons expressing VIP in layer 2/3 of wS1 during active sensing. We recorded across a broad range of neuronal cell classes present in layer 2/3 of wS1. Based on genetic markers, most layer 2/3 wS1 neurons can be classified as excitatory, inhibitory expressing PV, inhibitory expressing SST, inhibitory expressing VIP, or inhibitory expressing 5HT3A receptors but not VIP (Lee et al., 2010), with each class of inhibitory neuron likely serving different functions (Kepecs and Fishell, 2014; Tremblay et al., 2016). We found nicotinic depolarization specifically in VIP neurons with little modulation of the other cell classes (Figures 1, 2, and 3). The highly specific excitation of VIP neurons by nicotinic input during whisking might result from a higher expression of nicotinic receptors in VIP neurons compared to other subtypes. Indeed, VIP neurons have been reported to prominently express non-α7 subtypes of nicotinic receptors (Arroyo et al., 2012), which are relatively slow, consistent with the relatively slow nicotinic V_m_ changes in VIP neurons we report here during behavior. The relative location of the nicotinic receptors and the release site might be important (Bennett et al., 2012), and it is also possible that cholinergic axons might specifically innervate VIP neurons, which could be investigated in future electron microscopy studies. It is also important to note that it is becoming increasingly clear that VIP neurons are not a homogeneous population, and, in future experiments, it will be important to better define subtypes of VIP neurons (Prönneke et al., 2015), perhaps through intersectional genetics (He et al., 2016), which could reveal subtypes of layer 2/3 VIP neurons specialized for nicotinic disinhibition.

In general, disinhibition could contribute to many different aspects of neocortical circuit function. Disinhibition mediated by VIP neurons inhibiting SST neurons is likely to profoundly influence dendritic integration in the distal layer 1 dendrites of pyramidal neurons, which are targeted by SST-expressing Martinotti cells. Consistent with this hypothesis, at the same time that VIP neurons become excited during whisking, SST neurons hyperpolarize, and calcium signals are apparent in distal dendrites of pyramidal neurons (Gentet et al., 2012). VIP-mediated disinhibition could therefore enhance the impact of glutamatergic input onto distal layer 1 dendrites of pyramidal neurons. Important long-range inputs arrive in layer 1 including from higher-order POm thalamus, motor cortex, and secondary somatosensory cortex. VIP-mediated disinhibition could gate the impact of long-range input upon wS1, perhaps playing important roles in attentional modulation, as well as top-down and motor-related control of sensorimotor processing, which might contribute to detection of whisker stimuli (Takahashi et al., 2016) and object localization (Ranganathan et al., 2018; Xu et al., 2012). Previous work found that running caused increased calcium signals in VIP neurons of mouse V1, apparently via nicotinic excitation (Fu et al., 2014). Here, we found that whisking excited VIP neurons in wS1 via nicotinic input (Figure 1), in addition to the previously reported glutamatergic input from motor cortex (Lee et al., 2013). If motor activity in general excites VIP neurons across the mouse cortex, this could result in large-scale disinhibition, perhaps underlying some aspects of the brain-wide increases in activity observed during motor output in mice (Musall et al., 2019; Stringer et al., 2019). It will also be important to investigate the neural circuit mechanisms driving enhanced and prolonged activity of basal cholinergic neurons projecting to wS1 during whisker sensory processing and whisking.

Disinhibition has also been suggested to play a prominent role in controlling cortical plasticity. Interestingly, for example, a nicotinic signal driving a disinhibitory circuit in auditory cortex was shown to be important in auditory fear learning (Letzkus et al., 2011). Recently, cholinergic signals were also linked to reward coding (Hangya et al., 2015). Cholinergic reward signals in the neocortex might thus contribute to reward-based learning, perhaps, at least in part, by driving activity in VIP neurons, thus inhibiting SST neurons and causing disinhibition of layer 1 dendrites of excitatory neurons. Excitatory input to layer 1 distal dendrites of pyramidal neurons can cause calcium spikes and burst firing (Larkum et al., 1999), as well as NMDA spikes (Schiller et al., 2000). These events could be important triggers of synaptic plasticity. Interestingly, manipulation of VIP neurons suggests that increased activity of VIP neurons enhances plasticity in adult mouse visual cortex (Fu et al., 2015). Furthermore, in wS1, disinhibition by VIP neurons has also been shown to gate the induction of synaptic plasticity (Williams and Holtmaat, 2019). In the future, it will therefore be of great interest to record and manipulate VIP neurons and nicotinic signaling during reward-based learning and execution of goal-directed behavior.

## STAR★methods

### Key resources table

**Table:** 

REAGENT or RESOURCE	SOURCE	IDENTIFIER
**Bacterial and virus strains**		

AAV-FLEX-GCaMP6s-mRuby	Rose et al., 2016	Addgene 68717-AAV1
AAV-DIO-ChR2	Gift from Karl Deisseroth	Addgene 20298-AAV5
AAV-SIO-stGtACR2	Mahn et al., 2018	Addgene 105677-AAV1

**Deposited data**		

Dataset and MATLAB analysis code	This paper	https://doi.org/10.5281/zenodo.4352900

**Experimental models: organisms/strains**		

Mouse: VIP-Cre	The Jackson Laboratory	JAX: 010908
Mouse: PV-Cre	The Jackson Laboratory	JAX: 008069
Mouse: SST-Cre	The Jackson Laboratory	JAX: 013044
Mouse: ChAT-Cre	The Jackson Laboratory	JAX: 031661
Mouse: LSL-tdTomato	The Jackson Laboratory	JAX: 007909
Mouse: ChAT-ChR2-YFP	The Jackson Laboratory	JAX: 014546
Mouse: 5HT3A-GFP	Mutant Mouse Resource & Research Centers	MGI: 3846657

**Software and algorithms**		

MATLAB	Mathworks	https://www.mathworks.com/

### Resource availability

#### Lead contact

Further information and requests for resources and reagents should be directed to and will be fulfilled by the Lead Contact, Carl Petersen (carl.petersen@epfl.ch).

#### Materials availability

This study did not generate new unique reagents.

#### Data and code availability

The complete dataset and MATLAB analysis code are freely available at the open access CERN Zenodo database https://doi.org/10.5281/zenodo.4352900.

### Experimental model and subject details

All procedures were approved by Swiss Federal Veterinary Office (License number VD1628) and were conducted in accordance with the Swiss guidelines for the use of research animals. Both male and female mice were used. The mice were 4-8 weeks old at the time of head-post implantation (see below). The following transgenic mouse lines were used in this study: VIP-Cre (JAX: 010908) (Taniguchi et al., 2011); 5HT3A-GFP (MGI: 3846657) (Gong et al., 2003); PV-Cre (JAX: 008069) (Hippenmeyer et al., 2005); SST-Cre (JAX: 013044) (Taniguchi et al., 2011); LSL-tdTomato (JAX: 007909) (Madisen et al., 2010); ChAT-ChR2-YFP (JAX: 014546) (Zhao et al., 2011); and ChAT-Cre (JAX: 031661) (Rossi et al., 2011). VIP neurons were visualized by red fluorescence in VIP-Cre mice crossed with LSL-tdTomato mice. 5HT3A-non-VIP neurons were visualized through green fluorescence and absence of red fluorescence in triple transgenic mice made by crossing 5HT3A-GFP, VIP-Cre and LSL-tdTomato mice. PV neurons were visualized through red fluorescence in PV-Cre mice crossed with LSL-tdTomato mice. SST neurons were visualized through red fluorescence in SST-Cre mice crossed with LSL-tdTomato mice. For cholinergic optogenetic stimulation combined with targeted recordings of genetically-defined GABAergic neurons we made the following triple transgenic mice: VIP-Cre, LSL-tdTomato and ChAT-ChR2-YFP; PV-Cre, LSL-tdTomato and ChAT-ChR2-YFP; and SST-Cre, LSL-tdTomato and ChAT-ChR2-YFP.

### Method details

#### Experimental design

This study did not involve randomization or blinding. We did not estimate sample-size before carrying out the study. No data or subjects were excluded from the analysis.

#### Surgery

Mice were anesthetized with 2 – 4% isoflurane in pure oxygen. Body temperature was monitored and kept at 37°C throughout the surgery with the help of a heating pad. An eye cream was applied over the eyes to prevent them from drying. Carprofen was injected intraperitoneally or subcutaneously (100 μl at 0.5 mg/ml or 100 μl at 1.5 mg/ml) for analgesia. As local analgesic, a mix of lidocaine and bupivacaine was injected below the scalp before any surgical intervention. As a general analgesic treatment, ibuprofen was given in the drinking water for three days after surgery. A povidone-iodine solution was used for skin disinfection before surgery. To access the dorsal cortex, a part of the scalp was removed with surgical scissors. The remaining connective tissue was removed with a scalpel blade. After disinfecting the skull and rinsing it with Ringer’s solution, it was dried with cotton buds. A layer of super glue was then applied and a custom-made head fixation implant was glued to the right hemisphere of the skull. The head implant was further secured with self-curing denture acrylic. The left hemisphere of the dorsal cortex was free of denture acrylic and only covered by a thin layer of super glue for optical access. Three days post-implantation, intrinsic optical signal imaging was performed to localize the C2 whisker representation in wS1 barrel cortex, as described previously (Ferezou et al., 2007). Briefly, a piezoelectric actuator was used to repeatedly stimulate the right C2 whisker under light isoflurane anesthesia. A localized increase in the absorption of red light (625 nm) in the somatosensory cortex during whisker stimulation indicated the location of the C2 barrel column.

#### Viral injections

For optogenetic activation or inactivation of VIP neurons, a ∼1.5 mm craniotomy was performed and either AAV-DIO-ChR2 (Addgene 20298-AAV5) or AAV-SIO-stGtACR2 (Addgene 105677-AAV1) (Mahn et al., 2018) was injected in the center of the C2 barrel column at 800 μm, 600 μm, 300 μm and 150 μm below the dura in VIP-Cre mice. In total 150 nL was delivered through a glass pipette with a 21 – 27 μm inner tip diameter. The craniotomy was then fully closed with a 3 mm coverslip and the expression was monitored over days by two-photon microscopy.

For imaging of cholinergic axons, a 3 mm craniotomy was performed covering both the injection site and the C2 whisker representation in wS1. With a 21 – 27 μm inner tip diameter glass pipette, 1.5 μL of AAV-FLEX-GCaMP6s-mRuby (Addgene 68717-AAV1) (Rose et al., 2016) was slowly injected over 15 min in the center of the basal forebrain of ChAT-Cre mice (AP, −0.5 mm; ML, 1.8 mm, DV, −4.5 mm). The craniotomy was then closed with a glass window consisting of two 3 mm coverslips and one 5 mm coverslip glued together with light curing glue. This cranial window was then fixed to the skull with light curing glue and dental acrylic.

#### Whole-cell recordings targeted through two-photon imaging during behavior

A small craniotomy (∼1.5 mm) centered on the C2 barrel column was made with a dental drill under anesthesia. The dura was gently removed, and the brain surface was covered with 4% agarose maintained at body temperature (37°C). For stability, a 3 mm coverslip was halved and glued on 2/3rd of the craniotomy to allow pipette access. The recording chamber was then filled with Ringer’s solution. For pharmacological experiments, the drugs were mixed into the agarose and the Ringer’s solution (CNQX 1 mM; D-APV 2 mM; mecamylamine 1 mM). Mice were allowed to recover at least 2 h before the start of the recording.

Whole-cell recordings were targeted to fluorescent neurons in layer 2/3 of the C2 barrel column in wS1 visualized with a two-photon microscope (Gentet et al., 2010). The scanning system of the custom-made two-photon microscope consisted of a galvo-resonance mirror pair (8 kHz CRS, Cambridge Technology, USA). Fluorescence was excited with a 940 nm laser beam coming from a tuneable infrared laser (InSight DeepSee, Spectra Physics - Newport, USA) and focused into the cortex with an Olympus 40x objective (40x/0.80 W LUMPLFLN, Japan). The acquisition and imaging hardware (NI PXIe-1073, NI PXIe-6341, National Instruments, USA) was controlled by a MATLAB-based software (ScanImage SI5) (Pologruto et al., 2003). Green (510/84 BrightLine HC, Semrock, USA) and red (607/70 BrightLine HC, Semrock, USA) fluorescence were detected using GaAsP photosensor modules (H10770PA-40, Hamamatsu, Japan) after being reflected by a dichroic mirror (FF705-Di01-25x36, Semrock, USA) and passing through an infrared blocker (760/SP HC BrightLine, Semrock, USA). Photocurrents were amplified (DHPCA-100, FEMTO, Germany) and digitized with A/D board (NI 5732 14-bit, NI PXIe-7961R, National Instruments, USA). Frame acquisition rate was 30 Hz with resolution of 512x512 pixels.

Whole-cell recording pipettes were filled with an intracellular solution containing (in mM): 135 K-gluconate, 4 KCl, 4 Mg-ATP, 10 Na_2_-phosphocreatine, 0.3 Na_3_-GTP, and 10 HEPES (pH 7.3, 280 mOsmol/l). To target tdTomato-labeled neurons 1-20 μM of Alexa488 was included in the pipette solution, and to target GFP-labeled neurons 1-20 μM of Alexa594 was added. Neurons were recorded in the current-clamp configuration using a Multi-clamp 700B amplifier (Molecular Devices). Borosilicate patch pipettes with resistance of 5-7 MΩ were used. Electrophysiological data were low-pass Bessel filtered at 10 kHz and digitized at 20 kHz with an ITC-18 acquisition board (Instrutech). Data acquisition routines were custom-made procedures written in IgorPro software (Wavemetrics). Recordings were obtained without injecting current and membrane potential measurements were not corrected for the liquid junction potential.

Whisker movements were filmed with a high-speed camera as previously described (Gentet et al., 2010) and the whisker angle was extracted by a custom-made ImageJ analysis script.

Whisker stimulation was delivered by a 1-ms magnetic pulse acting on a metal particle attached to the C2 whisker (Figures 2 and 4) (Yamashita et al., 2013) or in some experiments to both B2 and C2 whisker (Figure 4). We did not find any significant difference between the results of stimulating the C2 whisker or both B2 and C2 whiskers, and the results were therefore pooled.

Optogenetic stimulation was performed by 10 ms (cholinergic axons) (Figure 3) or 500 ms (VIP neurons) (Figure 4) continuous illumination of the cortex with a 400 μm diameter optic fiber (Thorlabs) coupled to a 473 nm laser.

### Quantification and statistical analysis

#### Analysis of membrane potential

To compute the changes in V_m_ at whisking onset (Figure 1), the time of the initiation of each whisking bout was identified by visual inspection, and the V_m_ was averaged aligned to each onset of whisking. The mean V_m_ around the peak of the evoked response (100-300 ms after whisking onset) was compared to the mean baseline V_m_ in a symmetrical time window (300-100 ms before whisking onset) to compute the amplitude of the response (Figure 1D).

To calculate the change in V_m_ upon whisker deflection (Figure 2), trials in which the whisker was not moving were first selected (< 0.9° standard deviation from 100 ms before the whisker stimulus to 200 ms after the whisker stimulus). The amplitude of the response was computed as the difference between the mean V_m_ 50-100 ms after the whisker stimulus and the mean V_m_ 100-50 ms before the whisker stimulus.

The effect of cholinergic optogenetic stimulation (Figure 3) was evaluated as the mean change in V_m_ 5-20 ms after the onset of the blue light pulse relative to 20-5 ms before the onset of the blue light pulse for all cell classes, except the SST neurons in which the response was delayed. The amplitude of the response for SST neurons was computed as the mean difference in V_m_ comparing 10-50 ms after with 50-10 ms before light onset.

The late inhibitory component of the whisker-deflection evoked sensory response in excitatory neurons was evaluated separately for Quiet and Whisking trials (Figure 4). Quiet trials were defined as those in which the whisker angle changed less than 0.9° standard deviation quantified from 100 ms before the whisker stimulus to 200 ms after the whisker stimulus. Quiet and Whisking trials were averaged separately aligned to the whisker stimulus time. The amplitude of the hyperpolarization was computed as the mean difference in V_m_ between the baseline (20-0 ms before whisker stimulus onset) and 20 ms centered on the maximum of the hyperpolarization of the grand-average response.

#### Analysis of axonal fluorescence

For axonal GCaMP6s imaging (Figure S1C), the dynamic mean value of the fluorescence was extracted from a ROI selected on both the green and red channels with ImageJ. Background neuropil fluorescence was subtracted and changes in green fluorescence were computed as ΔF/F_0_. The red channel did not show whisking related changes in fluorescence.

#### Statistics

Data are represented in scatter-plots as mean ± SD; grand-average traces are presented as mean ± SEM after baseline subtraction. Comparison between cell types was performed using a Kruskal-Wallis test followed by pairwise comparison using Wilcoxon rank-sum tests with Bonferroni correction for the number of tests performed. Wilcoxon signed-rank test was used to assess significance in paired comparisons. The statistical tests used and the n numbers are reported explicitly in the main text, figures and figure legends.

## Supplemental information

Document S1. Figures S1 and S2

Document S2. Article plus Supplemental Information
