# Supplementary material for: Cell-type-specific nicotinic input disinhibits mouse barrel cortex during active sensing
Source: Neuron. 2021 Mar 3;109(5):778–787.e3. doi: 10.1016/j.neuron.2020.12.018 (PMC7927912; doi:10.1016/j.neuron.2020.12.018)
Supplement: Document S1. Figures S1 and S2 [file mmc1.pdf]

Neuron, Volume *109*

Supplemental information

Cell-type-specific nicotinic input disinhibits mouse barrel cortex during active sensing

Célia Gasselin, Benoît Hohl, Arthur Vernet, Sylvain Crochet, and Carl C.H. Petersen

Supplemental Information

Cell-type-specific nicotinic input disinhibits mouse barrel cortex during active sensing

Célia Gasselin, Benoît Hohl, Arthur Vernet,
Sylvain Crochet and Carl Petersen

Supplemental Information consists of:

Supplemental Figure S1, related to Figure 1

Supplemental Figure S2, related to Figure 4

Supplemental Figure S1

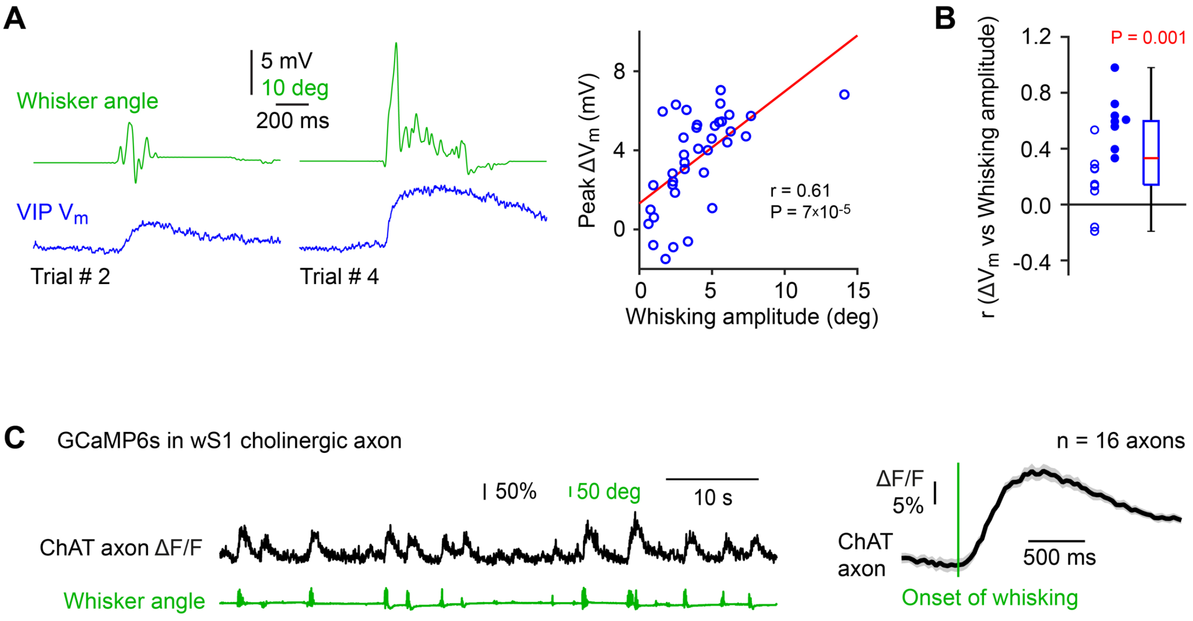

Supplemental Figure S1. The whisking-related depolarization of VIP neurons in the presence of CNQX & APV correlates with the strength of whisking, and whisking is accompanied by increased calcium in nucleus basalis cholinergic axons imaged in wS1, Related to Figure 1.

(A) Two whisking bouts recorded from the same VIP neuron (left). The whisker angle (top, green) and corresponding V_m (bottom, blue) are shown for whisking episodes (Trials) #2 and #4. For this neuron, the amplitude of the V_m response was significantly correlated to the mean amplitude of the whisker angle (SD of the whisker angle) across whisking bouts in this neuron (n = 37 whisking bouts, Pearson correlation r = 0.61 with t statistic P = 7×10^{-5}) (right).

(B) Analyzed across 17 VIP neurons in which we could record more than 10 whisking bouts, we found that the amplitude of the response of VIP neurons was significantly correlated to the amplitude of whisker angle in 8 neurons. Filled data points show neurons that were significantly correlated, whereas open data points were not significantly correlated. The coefficient of correlation (r) was significantly above zero at the population level (n = 17 neurons, Wilcoxon signed rank test, H_0 = median distribution of r not different from 0, P = 0.001).

(C) An example fluorescence trace from a GCaMP6s expressing cholinergic axon from nucleus basalis imaged in wS1 (left). With every bout of whisking, GCaMP6s fluorescence increases in this example axon (left). Averaged across all bouts of whisking (aligned at their onset) for each axon and further averaged across all imaged axons, the fluorescence shows an obvious increase following the onset of whisking (right). The thick trace shows the grand average $\Delta F/F_0$ across 16 axons, and the shading indicates SEM.

Supplemental Figure S2

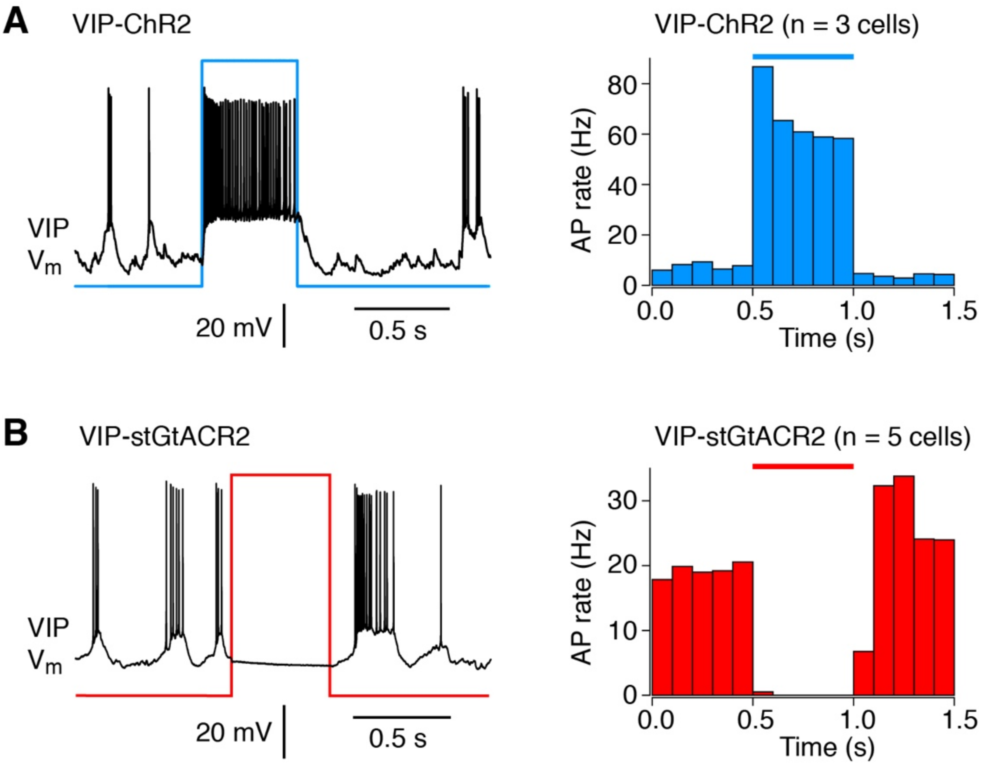

Supplemental Figure S2. Optogenetic excitation and inhibition of VIP neurons in layer 2/3 of wS1 in awake mice, Related to Figure 4.

(A) An example V_m recording from a VIP neuron expressing ChR2 (left). When blue light was applied to the cortex for 500 ms, the VIP neuron depolarized and increased action potential firing rate (left). Averaged across 500-ms blue light applications and further averaged across 3 recorded VIP neurons expressing ChR2, a clear increase in action potential firing was observed upon the optogenetic excitation (right).

(B) An example V_m recording from a VIP neuron expressing stGtACR2 (left). Spontaneous V_m depolarizations were absent during the 500-ms blue light pulse and no action potentials were fired (left). The action potential firing rate was computed across trials for each cell and further averaged across the 5 recorded neurons, showing a near-complete suppression of firing in VIP neurons during the optogenetic inhibition (right).
